# Modelling the dynamic relationship between spread of infection and observed crowd movement patterns at large scale events

**DOI:** 10.1038/s41598-022-19081-z

**Published:** 2022-09-01

**Authors:** Philip Rutten, Michael H. Lees, Sander Klous, Hans Heesterbeek, Peter M. A. Sloot

**Affiliations:** 1 Informatics Institute, University of Amsterdam, Amsterdam, The Netherlands; 2 Department of Population Health Sciences, Utrecht University, Utrecht, Netherlands

**Keywords:** Computational science, Infectious diseases

## Abstract

Understanding how contact patterns arise from crowd movement is crucial for assessing the spread of infection at mass gathering events. Here we study contact patterns from Wi-Fi mobility data of large sports and entertainment events in the Johan Cruijff ArenA stadium in Amsterdam. We show that crowd movement behaviour at mass gathering events is not homogeneous in time, but naturally consists of alternating periods of movement and rest. As a result, contact duration distributions are heavy-tailed, an observation which is not explained by models assuming that pedestrian contacts are analogous to collisions in the kinetic gas model. We investigate the effect of heavy-tailed contact duration patterns on the spread of infection using various random walk models. We show how different types of intermittent movement behaviour interact with a time-dependent infection probability. Our results point to the existence of a crossover point where increased contact duration presents a higher level of transmission risk than increasing the number of contacts. In addition, we show that different types of intermittent movement behaviour give rise to different mass-action kinetics, but also show that neither one of two mass-action mechanisms uniquely describes events.

## Introduction

Understanding how contact patterns arise from crowd movement is crucial for assessing the risk presented by mass gatherings for the spread of infectious diseases. The cancellation of mass gathering events was part of the unprecedented social distancing measures taken by governments worldwide, in response to the coronavirus disease 2019 (COVID-19) pandemic^1^. Examples of mass gatherings are large sporting, religious, and musical events in public sites. Although the risk of infection spreading at mass gatherings is generally recognised^2^, there is as yet no clear evidence-based understanding of the risk presented by these events^3^. Data-driven epidemiological models that estimate spread of infection at mass gatherings can help build a comprehensive quantitative framework for risk assessment^4,5^. Such models can evaluate benefits of mitigation strategies, assess the risk involved with lifting restrictions, and inform effective planning of mass gathering events^6–8^.

The transmission risk presented by mass gatherings depends on the specific contact patterns that arise from crowd movements during events. How contact patterns arise in dense crowds remains largely unexplored^9,10^. Epidemic models used in studies of disease spreading are typically based on the assumption of homogeneous mixing (see^11^ for a detailed exposition). Steps toward refinement of these assumptions have come from various directions, mostly driven by the availability of large data sets containing detailed information from mobility and communication networks, such as records from mobile phone users^12^. Network studies in particular have investigated how disease spread is affected by the connectivity patterns which characterise social networks^13,14^. Indeed, contact patterns due to any kind of social interaction can be represented as networks. Contact networks based on physical proximity data, and the dynamic nature of these networks, have gained increased attention recently^15–19^. However, proximity data is not easy to obtain and experiments so far have not included dense crowds at large events. In parallel, a small number of studies have investigated the direct effect of different movement models on mass-action assumptions. Rhodes and Anderson (2008) examined the contact rates resulting from a kinetic model of mobile individuals, and found that their results were in good agreement with homogeneously mixing mass-action models^20^. Hu et al. investigated the scaling of contact rates with population density, and proposed a non-linear function to describe the increase of contact rates from lower to higher densities^9^. Buscarino et al. investigated the effect of different movement models on epidemic spreading by analysing the dynamic contact networks arising from them^21^. More recently, Fofana and Hurford reviewed the effects of several common animal movement models on contact rates and epidemic spread^22^. They found that infection rates from different movement models, such as simple (uncorrelated) random walks or Lévy walks, do not deviate from mass-action assumptions, and do not significantly impact epidemic thresholds.

None of the studies described above specifically target pedestrian movement or crowd dynamics during mass gatherings, which may partially be explained by a lack of appropriate data available for scientific analysis. These studies, though not always explicitly, focus on human mobility involving travel by various transportation modes, and are not strictly confined to pedestrian movement. To date, the few studies which specifically address contact patterns in dense crowds are based on the decreasing relationship of pedestrian velocity with crowd density (e.g.^23^), which makes the contact patterns analogous to collisions in the kinetic gas model, as described in^9^.

What all the studies described above have in common is that they approach the problem of modelling contact patterns corresponding to a specific scenario (not necessarily mass gatherings), using a single type of movement model which is homogeneous in time. However, real life mass gathering events consist of multiple phases and conditions, which exist simultaneously or occur in chronological order. Every mass gathering event can be characterised as occurring in a number of distinct phases such as ingress or crowd gathering, dwell times, and egress. During these phases fundamental crowd conditions, such as density, change. Human movement behaviour is not homogeneous in time, and consists of changing behavioural modes^24^. People are not expected to move continuously, and individual movement behaviour naturally consists of alternating periods of movement and rest. This kind of intermittent movement behaviour also typically occurs during large crowded events that span long time periods^25,26^. Crowds at large events exhibit different types of movement behaviour, possibly corresponding to the different phases of the event^27^. Different types of movement behaviour present different levels of transmission risk. Depending on the disease in question, changing contact patterns will make different phases of an event more or less dangerous. Including these aspects in quantitative models is important if we aim for a realistic assessment and understanding of the way infection can spread during mass gathering events.

Here we study contact patterns from crowd movement data from large sports and entertainment events in the Johan Cruijff ArenA stadium in Amsterdam. We use localization of smart phones based on Wi-Fi detections to reconstruct individual trajectories, and use this as a proxy for human movement. We focus on the complex interplay between intermittent movement behaviour, consisting of alternating periods of movement and rest, and infection transmission. We build on previous work, in which we characterised the motion patterns, such as the intermittent pattern of movement and rest and corresponding waiting time distributions^25,26^. Throughout this paper we compare two events representing two types of collective crowd movement and corresponding forms of intermittency. We derive weighted contact networks from the collective trajectories, which are aggregated over the course of the event. These weighted networks have heavy-tailed weight distributions, which is not consistent with the gas kinetic model for contact patterns in dense crowds. Using random walk models, we demonstrate that the heavy-tailed weight distributions are explained by intermittent movement behaviour. Using simulation, we investigate how the different types of intermittent movement behaviour interact differently with a time-dependent infection probability. The longitudinal nature of the data sets allows us to track important measures such as incidence rates throughout the duration of the events. We show that the different types of intermittent crowd movement (in the two events) give rise to different mass-action kinetics. We show that mass gathering events are predominantly characterised by one of two mass-action mechanisms, but we also show that they are not uniquely described by either one of them. Taken together, our findings suggest that there is a need to revisit our assumptions about the relation between crowd behaviour and the transmission of infectious diseases. We propose an important refinement, and show the way forward to fill the gap in our understanding of pathogens spreading at mass gathering events.

We focus our analyses on the spread of an infectious disease that is directly transmitted during close contact or in physical proximity by droplets or aerosol transport, due to speech or inhalation of exhaled breath. The stadium consists of spaces with different dimensions, ventilation rates, air filtration, et cetera, which are expected to influence the probability of infection transmission in very large or outdoor spaces^28^. However, to isolate the effect of movement patterns on transmission, we regard the stadium as one continuous space with homogeneous conditions. We assume an incubation period which is longer than the timespan of the events we examine, so that secondary infections (i.e. individuals passing on the infection after being infected) play no role. These conditions correspond to many influenza-like diseases and match the virus transmission of SARS-CoV-2. The focus on primary infections limits the importance of contact network structure for the transmission dynamics. Network structure becomes relevant once we take spreading paths (through the network) into account. Therefore, network topology, as well as the time-order of contacts represented by a temporal network, are of less importance here, and we show contact networks only to convey some basic properties of the contact patterns.

We analyse two events, namely an ‘Eredivisie’ (premier division) football match, and a large dance event with DJ show. These two events represent two different levels of regulation of collective crowd movement. For a football match, movement is mostly regulated: during the episodes of the match people sit and watch, and after the match they leave the building. In contrast, for the dance event people were free to walk around the stadium, including the pitch. The event lasted more than 6 h, and the only important external drivers were the start and end of the DJ show, which lasted approximately 4 h. We expect that movement during the dance event is based on individual decisions about when (and where) to move. We expect statistical properties of the motion patterns to be emergent rather than imposed. We reproduce both forms of intermittency, regulated and individual, in random walk models, and investigate their effect on disease spread.

The rest of the paper is organized as follows. In Section “Data” we describe the data sets, preprocessing and translation to movement tracks, and the selection of devices. In Section “Movement track analysis we extract characteristics from the movement data which we use for random walk simulation models. In Section “Aggregated contact networks” we show several key properties of the aggregated contact networks from the movement data. In Section “Random walk models” we reproduce the observed characteristics of the contact networks using random walk models, and investigate their impact on the spread of infection. In Section “Transmission over the network” we show the time evolution of several important measures throughout the event, and in Section “Discussion” we discuss the findings.

## Data

We analyse movement data derived from Wi-Fi detections in the Johan Cruijff Arena stadium in Amsterdam. In previous work we have described various data and methods used to analyse the movement tracks obtained in this stadium, details of these can be found in our previous papers^25,26^. In those papers we analyse spatial and temporal aspects of the collective motion patterns. In this study we use the same methods as described in our most recent work^26^ and we therefore refer the reader to that reference for a detailed description of the data processing and movement track reconstruction. Here, we provide a brief overview.

**Figure 1:**
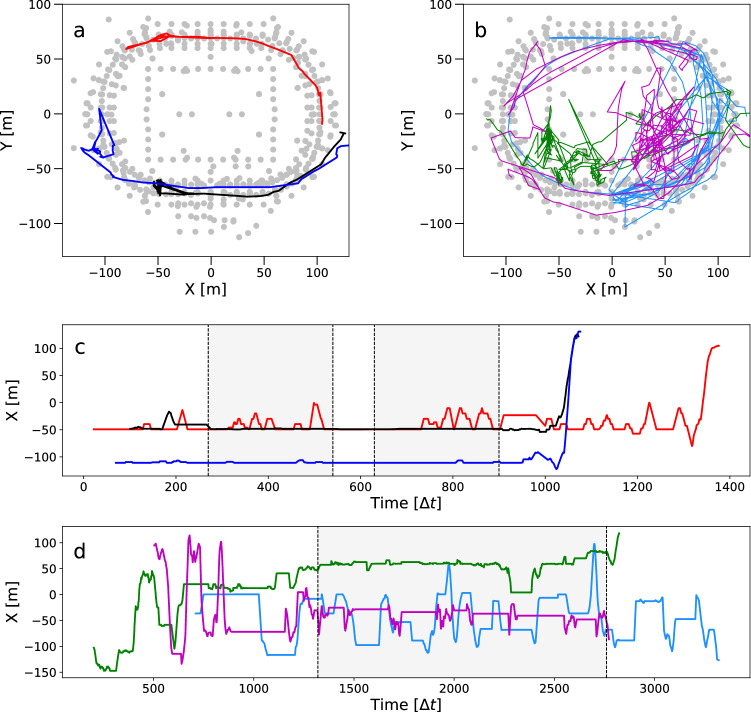
Examples of movement tracks during (**a**) the football match Ajax–Feyenoord, and (**b**) the Armin van Buuren dance event. (**c**, **d**) The same movement tracks as 1D time series of projections on the x-axis. Colors represent different individuals. Grey areas between dotted vertical lines indicate start and end times of (**c**) the football match, and (**d**) the DJ show. This figure is adopted from our previous work^26^.

The wireless network in the stadium consists of nearly 600 access points (APs) with known spatial coordinates. We estimate locations of anonymised smart phones using proximity detection^29^. At regular time intervals $\Delta t=10$ s we determine the position of a device at one of the APs and generate temporal sequences of AP locations. Drawing a line between successive locations produces a trajectory, or movement track. To deal with the considerable amount of fluctuation due to noise, we use a simple moving average to smooth the movement tracks (see Fig. 1 for examples). We ignore the *z*-coordinate and simplify the analysis to two-dimensions.

We restrict our analyses to movement tracks that fulfil certain requirements. When someone is not using his/her smartphone, the device eventually pauses all wireless communication. Therefore detection periods of devices alternate with periods without any detection. First we select devices whose detection periods span a minimum length of time, for which we use the duration of the main show of the events. For the football match the main show is the match, while for the dance event this was the DJ show. We select movement tracks with a minimum amount of detection gaps. We close the detection gaps using the interpolation method used by Rhee et al.^30^. We filter out devices that did not move at all during the whole event, as these likely represent static devices and not mobile phones.

Due to the gappy nature of the data, applying these criteria drastically reduces the number of devices that allow tracking. After filtering we have movement tracks of 362 devices of the football match, and 1048 devices of the dance event.

## Movement track analysis

Movement tracks of selected devices are characterised by an intermittent movement pattern. Periods of rest (‘waiting times’) alternate with episodes of movement that form larger displacements. This aspect of the movement data shows up clearly in the 1D projections of the movement tracks onto the x- and y-axes (see Fig. 1 for examples). The observation also agrees with our intuition about human behaviour. People stay in one place for some time, and then decide to change location, usually in one continuous movement bout. During the movement episodes individuals move with some degree of directional persistence. We have characterised these aspects in our previous works^25,26^.

The intermittent movement pattern is present in the movement data of both events. However, the reason behind the intermittent pattern is different in both events. For the football match, movement is mostly regulated and has a predictable character. We expect a typical movement trajectory that will remain mostly within a small, bounded region, and with larger displacements only at ingress, half time, and egress. For the dance event on the other hand, movement is based on individual decisions about when (and where) to move. We expect a pattern of multiple waiting times at different locations, and with variable duration. In Fig. 1 we show examples of both types of movement track from each event that agree with this intuition.

**Figure 2:**
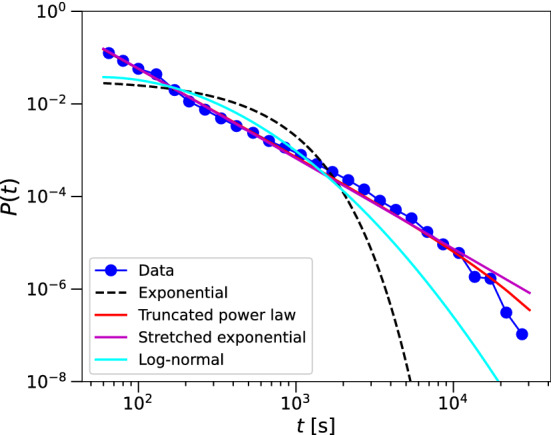
Probability distribution function of the waiting times on log–log scale, together with the MLE fits of the exponential, truncated power law, log-normal, and stretched exponential distributions. Model selection shows that the power law with exponent $\alpha =1.89$ provides the best description (see Appendix “Statistical methods and model selection” for details).

The accuracy of the movement tracks is too low for a meaningful spatial analysis of the contact patterns. However, the movement data demonstrate important properties of movement patterns in mass gathering events, namely intermittency and persistence. To study the exact effect of the movement patterns on the transmission of infection, we reproduce these properties in simulations (see Section “Random walk models”). In^25^ we show that individuals move with directional persistence and superdiffusively up to a scale set by the size of the stadium. It is this aspect of the motion that we wish to reproduce in simulations with dimensionless parameters (i.e. that do not mimic the spatial properties of the stadium). In Section “Random walk models” we explain how we do this. To simulate the intermittent movement patterns of the dance event we need to correctly characterise the waiting times. We extract waiting times from the movement tracks using a method similar to Boyer et al.^31^. (Note that we have investigated waiting times more extensively in our previous work^26^. To make the current research self-contained we repeat part of the analyses here.) We discretise the stadium into square cells of size $10\times 10$ m, a size that roughly corresponds to the measurement error. Waiting times *t* are measured as the number of consecutive time intervals ($\Delta t_i$) in the same grid cell. In Fig. 2 we show the distribution of waiting times on log–log scales. The data approximate a straight line on log–log scales, which indicates the waiting times are heavy-tailed. We fit statistical distributions to the measured waiting times, using maximum likelihood estimation (MLE) methods. We determine whether the data are better fit by an exponential distribution, a truncated power law, a log-normal, or stretched exponential distribution (see Appendix “Statistical methods and model selection” for details of the models and the MLE fitting). The exponential distribution is an indication of waiting times following a Poisson process. There have been a number of reports showing evidence that waiting times in the movement patterns of various animals (e.g.^31,32^), and also humans^30^, follow power law distributions. In our case, the waiting times are limited by the duration of the event and can only be reasonably identified as a truncated power law. The stretched exponential and log-normal distributions are models commonly used to describe heavy-tailed phenomena in complex systems^33^. Model selection based on Akaike’s information criterion (AIC)^51^ indicates that the most appropriate model is the truncated power law (see Appendix “Statistical methods and model selection” for details). The MLE value of the exponent $\alpha =1.89$ (95% CI 1.8844–1.9028).

## Aggregated contact networks

**Figure 3:**
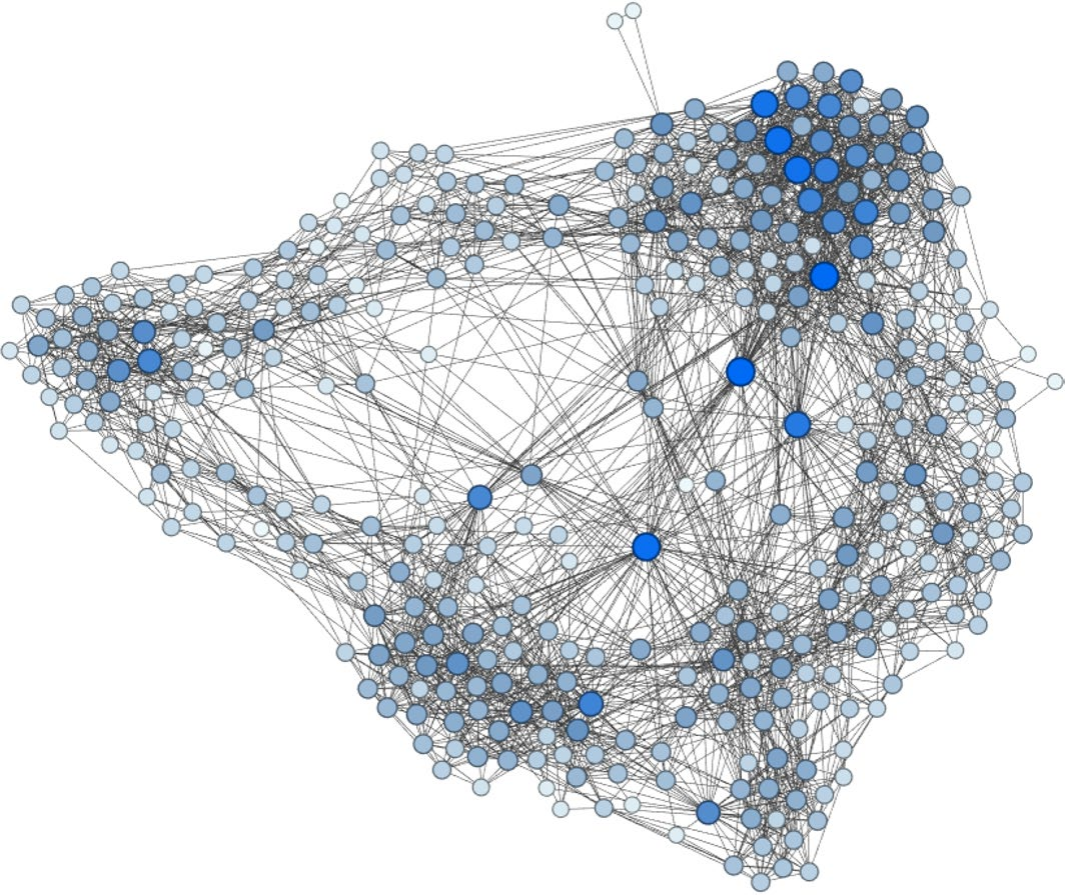
Visualisation of the aggregated network of the football match Ajax–Feyenoord. Color and size of nodes correspond to their degree.

We build contact networks by checking, at each time step, whether the pairwise proximity between devices *i* and *j* falls within a threshold distance (radius) $r=1.5$ m. We build a network aggregated over the whole event, which implies that any two individuals who are within a distance $<r$ in at least one time interval, have an edge between them in the network. In Fig. 3 we show a visualisation of the network of the football match Ajax-Feyenoord.

**Figure 4:**
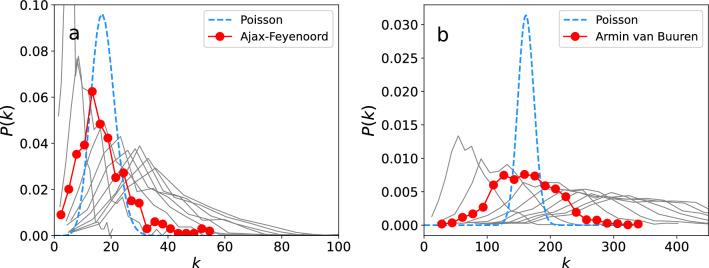
Degree distributions *P*(*k*) (red) together with Poisson predictions (blue), of (**a**) the football match Ajax–Feyenoord, and (**b**) the Armin van Buuren dance event, using threshold distance $r=1.5$ m. Grey lines illustrate the sensitivity of *P*(*k*) to *r*, and represent degree distributions in the range $r=0.5,\,1.0,...,\,5.0$ m.

The networks of both the football match and dance event are dense, and have small-world properties^34^. In Fig. 4 we show the empirical degree distributions, together with Poisson distributions

$$\begin{aligned} p(k)\sim e^{-\langle k\rangle }\frac{\langle k\rangle ^k}{k!} \end{aligned}$$

where $\langle k\rangle$ is the average node degree. The degree distributions are not heavy-tailed but, particularly for the dance event, the variance is much larger than the variance of the Poisson prediction. The observation that the degree distributions are not heavy-tailed suggests that ‘superspreaders’, interpreted here as individuals with relatively many different contacts, are possibly rarer than one would expect at such an event. However, this observation is based solely on the analysis of movement patterns and does not take into account other possible aspects giving rise to superspreading, such as heterogeneity in the infectiousness of individuals.

We compare the networks to random graphs of the same size *N* and average degree $\langle k\rangle$. The graphs are Erdős-Rényi models *G*(*N*, *p*), in which each pair of nodes is connected with probability $p=\langle k\rangle /(N-1)$^13^. We see that the empirical networks show the small-world phenomenon $L\geqslant L_{\text {random}}$ and $C\gg C_{\text {random}}$, especially for the football match (see Table 1).

**Table 1:** Average path length *L* and cluster coefficient *C* of the two events, compared to random graphs of the same size *N* and average degree $\langle k\rangle$.

	$L_{\text {actual}}$	$L_{\text {random}}$	$C_{\text {actual}}$	$C_{\text {random}}$
Ajax–Feyenoord	2.814	2.37	0.401	0.048
Armin van Buuren	1.852	1.846	0.355	0.154

The values shown in the table are averages over 20 random realisations.

The aggregated networks highlight an important difference with the assumptions of the existing model for dense crowds^9^, which proposes that pedestrian contacts are analogous to the collision process in the kinetic gas model. According to this model, when density increases, movement becomes increasingly impeded, and contacts are restricted to nearby individuals. Thus, contacts are expected to be local, which gives rise to lattice-like contact networks, without small-world properties. Clearly, these assumptions are valid within a sufficiently short time frame during an event. The model breaks down at the time scale of the whole event. During the phases of an event when crowd density is not at peak conditions, pedestrians are free to make larger displacements without difficulty. In addition, even during high density conditions individuals may relocate to visit bars, toilets, et cetera. Therefore, any crowd movement model in a bounded space and over longer time periods will give rise to a contact network with small-world properties.

**Figure 5:**
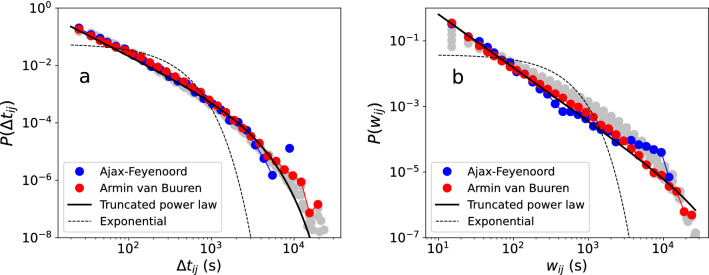
(**a**) Contact durations. (**b**) Weight distributions of the aggregated contact networks of the Armin van Buuren dance event (red), and the football match Ajax-Feyenoord (blue), on log–log scales. We show the MLE fits of the best models for the dance event according to model selection (see Appendix “Statistical methods and model selection” for details). We also show the distributions of contact duration and weights of the dance event for a range of values from $r=2$ to 10 m (grey).

Another important property which does not directly emerge from the kinetic gas model, is revealed when we look at temporal aspects of the interactions. The longitudinal nature of the data allows us to record for each pair of individuals *i* and *j*, a sequence of time intervals in which interaction occurred. A list of such sequences constitutes a temporal network^17^. From the contact event list we extract two measures of the interaction dynamics, namely the distributions of (1) contact duration $\Delta t_{ij}$ between *i* and *j*, and (2) weights $w_{ij}$, which represent the total contact time between *i* and *j* during the event. We measure pairwise contact duration $\Delta t_{ij}$ as continuous periods of physical proximity, where we simply merge and count the number of time intervals (in which physical proximity was detected) that are directly consecutive (zero gap length between them). In Fig. 5a we show the empirical probability distributions of contact duration $P(\Delta t_{ij})$ of both the football match and dance event on log–log scales. We see that the data of both events have broad tails, which indicates that the contact duration is heterogeneously distributed. The two distributions are also very similar, despite differences in the underlying event types and corresponding crowd dynamics. In Fig. 5b we show the distribution of weights $P(w_{ij})$ on log–log scales. We see that distributions of both events are heavy-tailed, and for a part decay with similar slope values. We apply model selection to the empirical probability distributions of the dance event, using the same model set as in Section “Movement track analysis” (see Fig. 2). This shows that the truncated power law provides the best fit for both contact duration and weights. The MLEs of the power law exponents are $\alpha =1.45$ (95% CI 1.4473–1.4593) for the contact duration, and $\alpha =1.63$ (95% CI 1.6202–1.6298) for the weights. In 5a and b we show only the exponential and truncated power law fits (see Appendix “Statistical methods and model selection” for details of the model selection). The distribution of measured contact duration may depend on the chosen value of distance threshold *r*. In Fig. 5a and b we show the distributions of contact duration and weights of the dance event for a range of values from $r=2$ to 10 m (grey lines). We see that the shape of the distributions is robust against variation in *r*. We note that the emergence of heavy-tailed contact duration distribution seems independent of the positioning accuracy of the underlying movement data. While we do not wish to propose a definite model for describing contact duration statistics in crowds, we expect heavy-tailed contact duration distributions to be the norm rather than the exception for mass gathering events. That the statistics of contact duration are robust across different settings has been observed before in social networks^15,35^, but it has not been demonstrated to characterise contact patterns in crowds. In the next section we propose an explanation for the heterogeneity in contact duration in terms of simple random walk models.

## Random walk models

In this section we investigate the effect of the empirically observed movement patterns on disease spreading dynamics. To do so, we reproduce the observed characteristics of the movement patterns in random walk (RW) models, and demonstrate the effect on infection transmission in simulations. The specific characteristics we reproduce are persistence and intermittency. To study their effects we first add different forms of intermittency to simple random walks, and next we create random walks which show both intermittency and persistence. We first illustrate the effect of these characteristics on the aggregated, weighted networks, and see whether we can reproduce the temporal features (as shown in Fig. 5). We run simulations of systems of random walkers and derive weighted contact networks as explained in Section “Aggregated contact networks”. We consider a system of $N=1000$ individuals for $T=500$ time steps. We use a threshold radius $r=1$ to define interaction by physical proximity. The individuals start at random positions in a $100 \times 100$ area, measured in units of the radius *r*. At each time step the individuals make a step of length 2, in a random direction taken from the uniform distribution on the interval $[-\pi ,\pi ]$. Note that these settings are similar to the simulations in^22^. All modifications we make to these settings (below), are parameterised to fit in with this framework.

To study the effect of intermittent movement behaviour, we divide each random walk into alternating periods of movement and rest. For the simulation of the regulated intermittency of the football match, we simply insert two collective breaks in the simulation, each lasting 100 time steps and starting at $t_1=50$ and $t_2=250$. To simulate the spontaneous, individual intermittency of the dance event, we take a stochastic approach. We divide each simulation time step in two parts. First, an individual draws a waiting time from a power law distribution $\phi (t)=t^{-\alpha }$, where $\alpha =1.9$, and $t\geqslant 5$. The parameter value $\alpha =1.9$ is as observed in the empirical data. The random walker remains at its current position for a number of $n=\lceil t\rceil$ steps. Next, an individual draws a ‘flight time’ from a stretched exponential distribution

$$\begin{aligned} f(\tau )=\beta \lambda \tau ^{\beta -1}\exp {\big (-\lambda (\tau ^{\beta }-a^{\beta })\big )} \end{aligned}$$

The flight times following the stretched exponential with stretching parameter $\beta =0.86$ are as empirically observed (see^25^). The parameter $\lambda$ controls the mean flight times. Larger flight times make the CTRW converge to the normal RW within the finite simulation time *T*. We show results for a range of values (see below). The random walker then takes a number of $n=\lceil \tau \rceil$ consecutive step lengths similar to the non-interrupted model described above. As these models are variations on the continuous-time random walk framework we refer to the regulated one as regCTRW, and the stochastic model as CTRW^36^.

**Figure 6:**
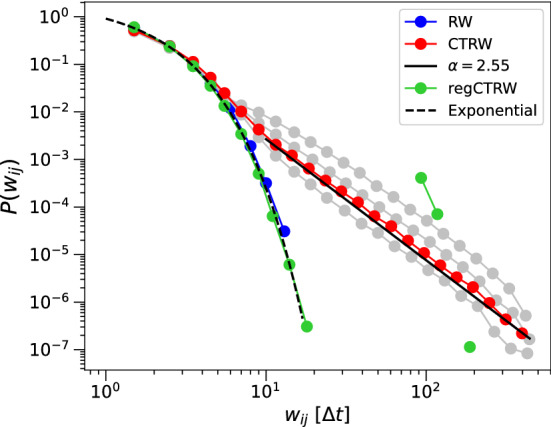
Weight distributions for the contact network from simulated random walkers, on log–log scales, of the random walk (RW)(blue), the RW interrupted by collective breaks (regCTRW)(green), and the stochastic CTRW (red). We show weight distributions of the CTRW for a range of values for $\lambda =0.1,\,0.05,\,0.033,\,0.02$ (grey). We show the MLE fit of an exponential distribution to the simple RW, and a power law to the weights of the CTRW with flight times using $\lambda =0.033$. MLEs are obtained using the Python powerlaw package^37^.

In Fig. 6 we show the resulting weight distributions of the simple RW, regCTRW, and CTRW. The simulated regCTRW weight distribution (green) shows a pronounced bimodal distribution (driven by the perfectly regulated collective breaks). We see that the weights of both the RW (blue) and regCTRW decay exponentially, but that the regCTRW distribution has a second peak at 100 time steps, which corresponds to the length of one break. A small number of individuals must have been in close proximity during both breaks, as there are observations at 200 time steps. The empirical weight distribution of the football match (Fig. 5b) shows a less pronounced bimodal distribution, but there is a clear peak in contact duration distribution (Fig. 5a) associated with the length of the match. The weights of the stochastic CTRW model (Fig. 6, red) follow a heavy-tailed distribution. We show weights of the CTRW for a range of values for $\lambda =0.1,\,0.05,\,0.033,\,0.02$. We show the MLE fit of an exponential distribution to the simple RW, and a power law $p(w)\sim w^{-\alpha }$ to the weights of the CTRW with flight times using $\lambda =0.033$. The MLE of the power law exponent $\alpha =2.55$. The resulting weight distribution of the CTRW resembles the empirically observed weight distributions as both are heavy-tailed distributions that can be described by a power law over a certain range (Fig. 5b). The exact forms of the distributions and relationship with the underlying movement models are out of scope for this paper.

Next we investigate the effect of the empirical movement patterns on the spread of infection. To do so, we need to reproduce both characteristics of the observed motion patterns, persistence and intermittency, in random walk simulations. In our previous work^25^ we have shown that during the movement episodes individuals do not diffuse randomly, but move with some degree of directional persistence and superdiffusively up to a scale set by the size of the stadium. Although these characteristics are Lévy-like, we did not find evidence of pure Lévy walk behaviour. In random walk models, persistence in direction is expressed through autocorrelation in turning angles between successive movement steps. This behaviour can be modelled using a correlated random walk (CRW)^38,39^. Angles between successive movement steps are sampled from the Von Mises distribution^40^. Again, we compare three RWs, where the first is the (continuously moving) CRW, the second is the CRW interrupted by collective breaks, and the third is a stochastic intermittent CRW. The waiting times are added to the models according to the mechanisms described above. As these models are now continuous-time correlated random walks we refer to them as regCTCRW, and CTCRW.

**Figure 7:**
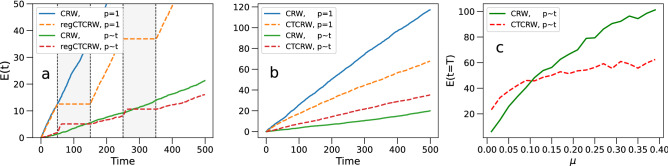
Time evolution of the number of exposed *E*(*t*) of the CRW and (**a**) the regCTCRW, and (**b**) the CTCRW, using $p_{\text {inf}}=1$, and $p_{\text {inf}}\sim t_{ij}$. (**c**) The final number of exposed $\langle E(t)\rangle$ at $T=500$ of the CRW and CTCRW, versus transmissibility parameter $\mu$ [in Eq. (1)]. All results are averaged over 30 simulations.

We compare the infection spreading dynamics of the three models (CRW, regCTCRW, and CTCRW). We focus on a basic susceptible-exposed (SE) model, which we run on top of the simulated random walks. Due to the short time span of the events we’re simulating, we ignore transitions $E\rightarrow I$, and recovery ($I\rightarrow R$). We divide the population in the three states susceptible (S), exposed (E), and infectious (I). At $t=0$ we introduce a number of infectious individuals $I_0$ (seeds), which remains constant throughout the simulation. If a susceptible agent interacts with an infectious agent, there is a probability $p_{\text {inf}}$ that the susceptible agent transfers to the exposed state. In Fig. 7 we compare the time evolution of the number of exposed *E*(*t*) of the CRW, with both (a) the regCTCRW, and (b) CTCRW, using $I_0=1$ and $p_{\text {inf}}=1$. Not surprisingly, the spreading stops completely during the breaks for the regCTCRW (Fig. 7a), and is slowed down for the CTCRW (Fig. 7b), due to agents effectively moving less, and having less encounters with other agents.

In reality, infection transmission requires prolonged contact between two individuals, instead of occurring instantaneously. We assume that the number of infectious units an individual is exposed to increases with contact duration, and that the risk of infection increases accordingly. To implement this, we introduce an infection probability as a function of contact duration, defined as

1 $$\begin{aligned} p_{\text {inf}}(t)=1-e^{-\mu \,t_{ij}} \end{aligned}$$

where $t_{ij}$ is the cumulative contact duration between individuals *i* and *j*, which is measured as the number of time steps spent in proximity, and $\mu$ is a parameter modulating the increase of $p_{\text {inf}}$ with $t_{ij}$. Equation (1) is based on a model proposed by Riley et al.^41^, and further developed in a number of publications (e.g. see^42–45^). Note that the time steps spent in close contact are not necessarily consecutive. Because this model produces lower numbers of exposed agents, we set $I_0=10$ to enhance the effect within the simulation time. In Figure 7 we show the time evolution of the number of exposed *E*(*t*) of the CRW and (a) the regCTCRW, and (b) the CTCRW, using $\mu =0.04$ in Eq. (1). The parameter value $\mu =0.04$ is arbitrarily chosen and serves illustrative purposes of the simulations. Below we explore further some of the implications of this parameter. We see that in this case (i.e. $p_{\text {inf}}\sim t_{ij}$), the number of exposed agents on the regCTCRW model (red dashed curve) jumps up at the start of each break (shaded grey areas), due to individuals having prolonged contacts with other agents. However, we also see that the curve rapidly saturates, due to exhaustion of the number of susceptibles near an infectious individual. Because of this, the spreading on the regCTCRW ends up producing lower numbers of exposed than the CRW. This shows that a stationary (e.g. seated) crowd imposes an increased infection risk if the infection probability is time-dependent, but that the total number of exposed individuals is bounded by the number of individuals in proximity.

In Fig. 7b we see that the CTCRW model produces higher numbers of exposed agents than the CRW. The infection spreading again benefits from agents occasionally not moving, and having prolonged contacts with other agents. However, due the random, non-overlapping periods of rest there are no saturation effects. This result points to the fact that, if the infection probability is time-dependent (i.e. $p_{\text {inf}}\sim t_{ij}$), there exists a crossover point where prolonged contacts present more risk than encountering new individuals. If the transmissibility [i.e. $\mu$ in Eq. (1)] becomes lower/higher disease transmission benefits more/less from longer contact duration. In Fig. 7c we show the dependency of the final number of exposed *E*(*t*) at $T=500$, produced by both the CRW and CTCRW, on the value of $\mu$ [in Eq. (1)]. We see a clear crossover where the two curves intersect and one model poses a higher level of transmission risk than the other. This shows that, if the infection probability is time-dependent, an intermittently moving but freely mixing crowd may present the highest level of transmission risk.

These results also show that the actual transmission risk imposed by different types of crowd movement (i.e. stationary/dynamic) depends on the infection probability. As real crowded events are expected to consist of mixtures of different crowd movement behaviours, estimating the risk involved is not straightforward.

## Transmission over the network

**Figure 8:**
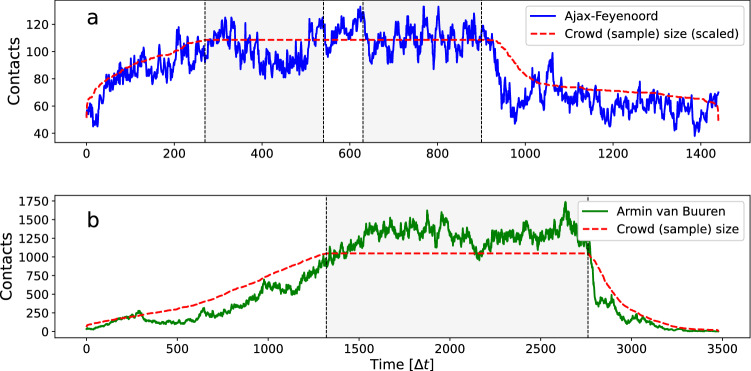
The number of contacts per time interval $\Delta t$, during (**a**) the football match Ajax–Feyenoord (blue), and (**b**) the Armin van Buuren dance event (green). Also shown are crowd sample size *N* versus time curves (red). Note that, as a visual aid, the crowd sample size curve of the football match in (**a**) is arbitrarily scaled so that it overlays the contacts curve (blue). The grey areas indicate start and end times of the match and DJ show.

**Figure 9:**
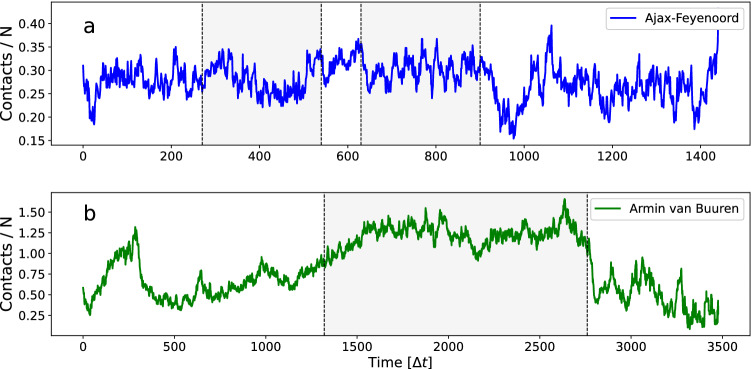
The number of contacts per time interval $\Delta t$ normalised by the crowd sample size *N*(*t*), during (**a**) the football match Ajax–Feyenoord (blue), and (**b**) the Armin van Buuren dance event (green). The grey areas indicate start and end times of the match and DJ show.

So far, we have studied the effect of two types of intermittent crowd movement (regulated and stochastic) on the spread of infection, in simulations with constant population density. Real life events occur in a number of distinct phases such as ingress, dwell times, and egress. During these phases the crowd density changes. In this section we show how the two types of intermittent crowd movement relate to crowd density and give rise to different mass-action kinetics. The data give information about how contact patterns are formed over time. More specifically, it gives information on *when* contacts occur in time. The longitudinal nature of the data set allows us to see when, and for how long, these contacts occur. Instead of aggregating the contacts and representing it as a static network (as in Section “Aggregated contact networks”), we look at the network in each separate time interval. We can refer to these networks as ‘time slices’. The number of edges in each time slice represents the number of contacts in the time interval. In Fig. 8 we show the time evolution of the number of contacts. Note that this is similar to ‘collisions’ within the gas kinetic framework (Cf.^9^). Tracking the contact rate over time enables us to study its dependence on the total crowd size, i.e. its effective mass-action kinetics. The contact curves can be seen to have a similar shape as the crowd size (*N*) curves (red lines in Fig. 8). We can visualise this aspect better by normalising the contacts using crowd size *N*(*t*), see Fig. 9. The contact curves can now both be seen as fluctuating around a constant value. This suggests contact rates increase linearly with crowd size or density. However, we also see that, especially in the case of the dance event, there is considerable fluctuation. This suggests that additional factors are required to explain variations in the contact patterns. We return to this issue at the end of this section.

**Figure 10:**
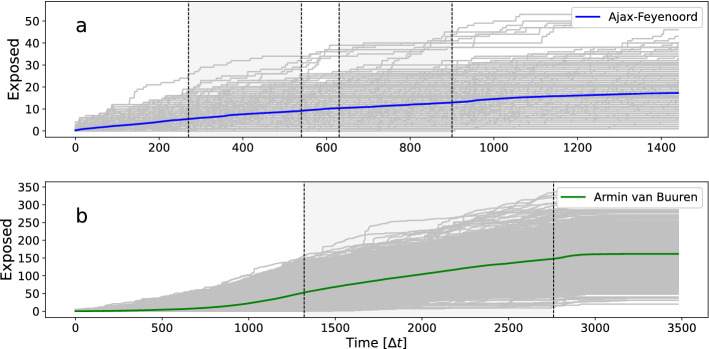
The cumulative number of exposed individuals per time interval $\Delta t$, during (**a**) the football match Ajax–Feyenoord (blue), and (**b**) the Armin van Buuren dance event (green). The grey areas indicate start and end times of the match and DJ show.

To explore transmission of infection based on the empirical contact patterns, we consider an SEIR model which we run on top of the measured contact networks. We divide the population in the three states susceptible (S), exposed (E), and infectious (I). Due to the short time span of the events, we ignore transitions $E\rightarrow I$, and recovery ($I\rightarrow R$). Therefore, secondary infections (i.e. individuals passing on the disease after being exposed) are not included. We randomly select an individual which we introduce as an infectious seed, while all other individuals are susceptible. We assume transmission takes place at first encounter (i.e $p_{\text {inf}}=1$), and when there is contact between the seed and a susceptible, the last one changes into the exposed (E) state. We record this event, and track the growth of the number of exposed individuals from one particular seed over time, which is the cumulative incidence curve. We run this process for each individual in the sample. In Fig. 10 we show for both events all individual incidence curves (grey), and also the means (blue and green). Note that the cumulative incidence curves are simply the growth of the node degrees *k* over time, and the mean is just the time evolution of the mean degree $\langle k\rangle$.

**Figure 11:**
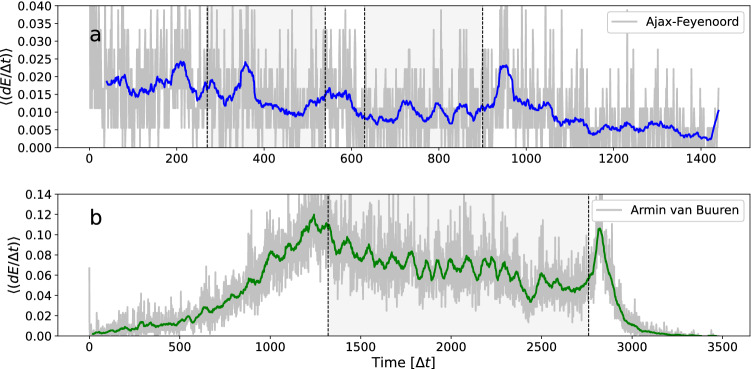
The number of newly exposed individuals per time interval $\Delta t$, during (**a**) the football match Ajax–Feyenoord (blue), and (**b**) the Armin van Buuren dance event (green). The grey areas indicate start and end times of the match and DJ show.

We are especially interested in possible variation in the growth rate of the incidence curves during the event. This is better visualised by recording the number of infectious contacts per time interval. In Fig. 11 we show incidence rate curves $\langle (dE/\Delta t)\rangle$, where the brackets $\langle ...\rangle$ denote averaging over all possible individuals in the sample. Note that the incidence curves in Fig. 10 are simply the integral over this. The incidence rate curves represent newly exposed individuals per time interval, where we assume disease transmission takes place at first encounter between an infectious and a susceptible individual (i.e $p_{\text {inf}}=1$). We see that both curves in Fig. 11 are fluctuating. For the football match the curve can be seen to fluctuate around a constant value, but it is slowly declining and then has large peak after the match. Note that this particular shape has no obvious relationship with density (see crowd sample curve in Fig. 8), and if at all, seems negatively correlated. For the dance event the curve has a more pronounced shape, with the largest peak occurring just before the start of the DJ show, and a second peak right after the end of the show.

It is interesting to evaluate Figs. 8, 9, 10 and 11 in relation to the two different approaches to mass-action assumptions of homogeneous mixing^9,20,46^. According to the density-dependent mechanism, the rate of increase of exposed individuals follows (within a SEIR framework) $dE/dt=\beta SI$, where $\beta$ is the transmission rate parameter. According to the frequency-dependent mechanism, the relation is $dE/dt=\beta SI/N$. For the density-dependent mechanism to hold, the spatial area must be constant so that an increase in population size is an increase in density. The frequency-dependent mechanism is not only more in agreement with our natural intuition, but also more supported by empirical evidence^20,47^.

Seen from this perspective, the results of the football match and the dance event paint two different pictures. Note that in the density-dependent regime the exposed rate curve should scale in a linear fashion with population density, while in the frequency-dependent regime the exposed rate curve should assume a constant value, independent of the crowd density. In Fig. 8 we see that contact patterns (collision frequency) evolve similarly as the crowd size curves (red dashed lines). This suggests contact rates increase linearly with crowd size, and both events are in the density-dependent regime. Figure 9 shows that this aspect is stronger for the football match, while the dance event shows more deviation from this rule. This distinction between the events is more pronounced in Fig. 11. In Fig. 11a we see that for the football match, *dE*/*dt*, as approximated by $\langle (dE/\Delta t)\rangle$, seems to fluctuate around a constant value, which indicates the transmission rate is in agreement with the frequency-dependent mechanism (i.e. $\sim \beta SI/N$), and independent of population size *N* or density. On the other hand, Fig. 11b shows that, for the dance event, *dE*/*dt* still closely resembles the crowd size curve *N*(*t*) (red dashed line in Fig. 8b). This suggests that at least for some part of the event, the transmission rate increases linearly with population size (i.e. $\sim \beta SI$), as formulated by the density-dependent mass-action law. These observations agree with our intuition as the area of the stadium is constant and an increase in crowd size means an increase in density. However, this effect is much less important for the football match, where the amount of mixing is constrained. Apart from entry and exit, people remain seated with a fixed number of individuals in close proximity, independent of the total crowd size. It would therefore be natural that contact patterns in the regulated crowd movements of the football match follow the frequency-dependent regime, while the contact patterns from the freely moving dance event visitors follow the density-dependent mechanism.

Nevertheless, in Figs. 8, 9, 10 and 11 we also see considerable deviation from both mass-action mechanisms. We see that, even at constant *N* (e.g. during the DJ show), contact rates and $\langle (dE/\Delta t)\rangle$ vary. For both events we see a slow decline of $\langle (dE/\Delta t)\rangle$ which might be explained by the exhaustion of susceptible individuals in someone’s proximity. However, in both events we see a large peak at the end of the event, despite a decrease in crowd size *N* at that point in time. This suggests that the transmission rate $\beta$ is not constant and varies over time, independent of *S*, *I*, or *N*. The transmission rate $\beta$ is the product of infection probability *p* and contact rate *C*, i.e. $\beta =p\,C$^47^. In the simulation analysis (i.e. Fig. 11) $p=1$ is constant, so it is the contact rate *C* which is time-varying. These observations support the idea that neither the density-dependent mechanism or the frequency-dependent mechanism uniquely describes the scaling of contact rates in relation to crowd density, and that in many situations the contact rate is a combination of the two extremes^9,20^. However, the results also suggest that, in addition to the two types of mass-action mechanisms, additional factors are required to explain variations in the contact patterns, and that these factors are related to changes in crowd movement behaviour during an event.

## Discussion

In this study we present several important features of the transmission of pathogens at large mass gathering events. These features, brought to light by enlarging the observational time frame, are illustrated by the temporally weighted contact networks, aggregated over the course of the event. First of all, the observed small-world property of the contact networks is not explained by the conceptual framework of existing contact models. According to this framework, contacts are restricted to nearby individuals, which gives rise to lattice-like contact networks, without small-world properties. This assumption is applicable only to a sufficiently short time frame. The fact that the contact networks fall somewhere between a lattice and a complete (fully connected) network indicates that pedestrian movements at events naturally consist of a mixture of local and long-distance displacements. This shows that movement behaviour during large events is not homogeneous in time, and key movement parameters are time-varying. Secondly, the aggregated contact networks have heavy-tailed contact duration distributions. We show that this observation is independent of the positioning accuracy of the underlying Wi-Fi-based movement data. Using simple random walk models we demonstrate the link between intermittent movement behaviour, consisting of alternating periods of movement and rest, and heavy-tailed contact duration distributions. Heterogeneously distributed contact duration is common to social networks but so far has not been demonstrated for crowds. We note that intermittent movement behaviour, and corresponding heterogeneously distributed contact duration, are expected to be the norm rather than the exception for mass gathering events.

Using simulation, we have shown how different types of intermittent movement behaviour interact with key infection processes, indicating that the temporal dynamics of the contact patterns strongly influence transmission dynamics at mass gatherings. Our results expose the existence of a crossover point where increased contact duration presents a higher level of transmission risk than increasing the number of contacts. Therefore, understanding the temporal dynamics of the underlying crowd movements as well as specific disease transmission characteristics are both essential for estimating transmission rates at mass gathering events.

To isolate the effect of movement patterns on pathogen transmission, we have made several simplifying assumptions, such as conditions being constant throughout the stadium, and only considering transmission during close contact or in physical proximity. In addition we have assumed key epidemiological parameters, such as infection probability and susceptibility, to be equal for all individuals. Both of these are certainly simplifications. Investigating the impact of heterogeneity in these factors on the spreading dynamics at crowded events are important opportunities for further research.

The longitudinal nature of the data set has allowed us to study how contact rates and incidence evolve in relation to crowd size. We have shown that different types of events give rise to one of two types of mass-action kinetics. We have also shown that contact rates and spreading on the empirical movements are time-varying. This suggests that contact rates are probably the result of a dynamic combination of the two mass-action mechanisms. During an event one of the two mechanisms may become more dominant due to changes in collective movement behaviour. However, even within one of the two mass-action frameworks, parameters are not necessarily constant, giving rise to even more variation in contact rates. For example, individual movement parameters such as velocity, step length and persistence may change over time, possibly in response to external factors. These results indicate that a detailed understanding of pedestrian movement behaviour in dense crowds is crucial for predicting the specific mass-action process that will emerge.

Our work shows the importance of a more integrative approach to mass gathering events, which considers these events in their full spatio-temporal complexity. Here we have taken a first step and show how different event scenarios, with different intermittent crowd behaviours, lead to different contact rates. A more holistic approach to events is essential if we want models to inform policy makers on specific types of events and the risks they present. It will also increase our understanding of the different phases during mass gathering events and the varying levels of risk they present in relation to the transmission characteristics of specific pathogens. This could ultimately serve event organisers in planning a safe return to normal operations as well as developing long-term risk protocols for large mass gatherings.

## Data Availability

The analysis of Wi-Fi detection data in the current study is licensed under a contractual agreement between the University of Amsterdam and the Johan Cruijff Innovation Amsterdam. Derived movement tracks, and all custom code used to analyse the data in this study, are freely available at: https://github.com/philiprn/Crowd-Epidemiology.

## Appendix: Statistical methods and model selection

We use statistical methods of Clauset et al.^48^ and Edwards et al.^49^ for fitting the distributions. We also check results using the Python powerlaw package^37^, which has implemented the methods from^48^.

### Models

We fit the following statistical distributions to various measured quantities:

- The exponential distribution, with probability density function defined as:
  2 $$\begin{aligned} p(t)=\lambda \exp {\big (-\lambda (t-a)\big )} \end{aligned}$$
- The truncated power law distribution distribution, with probability density function defined as:
  3 $$\begin{aligned} p(t)=(t+a)^{-\alpha }\text {e}^{-t/t_c} \end{aligned}$$
- The stretched exponential distribution, with probability density function defined as:
  4 $$\begin{aligned} p(t)=\beta \lambda t^{\beta -1}\exp {\big (-\lambda (t^{\beta }-a^{\beta })\big )} \end{aligned}$$
- The log-normal distribution, with probability density function defined as:
  5 $$\begin{aligned} p(t)=\frac{1}{t\sigma \sqrt{2\pi }}\exp {\Big (-\frac{ (\log {t}-\mu )^2}{2\sigma ^2}\Big )} \end{aligned}$$
  where $t_c$ is the exponential cutoff value, and *a* is the lower bound of the fitting range.

### Maximum likelihood estimation

The maximum likelihood estimate (MLE) of the parameter $\lambda$ of the exponential distribution $p(x)=\lambda e^{-\lambda (x-a)}$ is given by

6 $$\begin{aligned} {\hat{\lambda }}=1\Big /\Big (\sum _{i=1}^{n} x_i/n-a\Big ) \end{aligned}$$

where *n* is the number of data points, and *a* is the lower bound of the fitting range. In this research *a* is loosely determined as the value after which the decay starts in the empirical frequency distribution.

There are no analytical solutions for the MLEs of the truncated power law distribution, stretched exponential, and log-normal distributions. In this case we numerically minimise the negative log-likelihood function

7 $$\begin{aligned} L(\theta )=-\sum ^{n}_{i=1}\log {p(x_i\big |\theta )} \end{aligned}$$

For the numerical minimisation we use Python library functions (following^37^).

### Confidence intervals of estimated parameters

To compute confidence intervals we use the likelihood profile method^50^. The method compares the likelihood of the MLE of a parameter $\theta$ with other values of that parameter. According to statistical theory

8 $$\begin{aligned} {\mathscr {R}}=2\big [L(\theta )-L(\theta _{\text {mle}})\big ] \end{aligned}$$

has a chi-square distribution with one degree of freedom. We can find 95% confidence interval boundaries by using the fact that $Pr\{\chi ^2 \leqslant 3.84\}=0.95$, and numerically solving $L(\theta )-L(\theta _{\text {mle}})=1.92$. For models with two parameters we perform the test for each parameter separately. We systematically vary the parameter of interest, and at each instance compute the value for the other parameter that maximises the likelihood at that point.

### Akaike model selection

To compute Akaike weights we need the Akaike Information Criterion (AIC)

9 $$\begin{aligned} \text {AIC}=2L(\theta _{\text {mle}})+2K \end{aligned}$$

for which we require the value of the negative log-likelihood function at the maximum (MLE), and where *K* is the number of parameters to be estimated^49,51^. The AIC differences are

10 $$\begin{aligned} \Delta _i=\text {AIC}_{\text {i}}-\text {AIC}_{\text {min}} \end{aligned}$$

where $\text {AIC}_{\text {min}}$ is the AIC of the model with the minimum AIC, which is considered as the best model. The Akaike weights are give by

11 $$\begin{aligned} w_i=\frac{\exp {(-\frac{1}{2}\Delta _i})}{\sum \limits _{m=1}^{M}\exp {(-\frac{1}{2}\Delta _m)}} \end{aligned}$$

where *M* is the set of models to be compared.

### Model selection results

*Waiting times* In Section “Movement track analysis” we fit the statistical models given in Appendix “Models” to the measured waiting times (see Fig. 2). In Table 2 we show model selection results based on Akaike weights, using $a=6\,\Delta t$ ($=60$ s). The truncated power law ($w_{\text {tpl}}=1$) provides the best description of the data.

**Table 2:** Overview of the model selection results for the measured waiting times (see Fig. 2), based on Akaike weights.

Model	MLE parameters	95% CI	$\Delta$AIC	Akaike weights
Exponential	$\lambda =0.02812$	(0.0279, 0.0284)	60,105	0
Truncated power law	$\alpha =1.8936$	(1.8844, 1.9028)	0	1
Log-normal	$\mu =2.8523$	(2.8426, 2.8621)	38,245	0
	$\sigma =1.0606$	(1.0537, 1.0675)		
Stretched exponential	$\lambda =108.02$	(108.01, 108.03)	247	0
	$\beta =0.0085$	(0.0085, 0.0085)		

*Contact duration* In Section “Aggregated contact networks” we fit the statistical models given in Appendix “Models” to the measured contact duration distribution (see Fig. 5a). In Table 3 we show model selection results based on Akaike weights, using $a=2\,\Delta t$ ($=20$ s). The truncated power law ($w_{\text {tpl}}=1$) provides the best description of the data.

**Table 3:** Overview of the model selection results for the measured contact duration (see Fig. 5a), based on Akaike weights.

Model	MLE parameters	95% CI	$\Delta$AIC	Akaike weights
Exponential	$\lambda =0.0518$	(0.0514, 0.0521)	69,852	0
Truncated power law	$\alpha =1.4533$	(1.4473, 1.4593)	0	1
Log-normal	$\mu =2.1575$	(2.1490, 2.1661)	39,884	0
	$\sigma =1.2074$	(1.2014, 1.2135)		
Stretched exponential	$\lambda =1.9569$	(1.9469, 1.9669)	1186	0
	$\beta =0.2220$	(0.2210, 0.2230)		

*Weights* In Section “Aggregated contact networks” we fit the statistical models given in Appendix “Models” to the measured weights (see Fig. 5b). In Table 4 we show model selection results based on Akaike weights, using $a=1\,\Delta t$ ($=10$ s). The truncated power law ($w_{\text {tpl}}=1$) provides the best description of the data.

**Table 4:** Overview of the model selection results for the measured weights (see Fig. 5b), based on Akaike weights.

Model	MLE parameters	95% CI	$\Delta$AIC	Akaike weights
Exponential	$\lambda =0.0367$	(0.0364, 0.0369)	234,745	0
Truncated power law	$\alpha =1.6250$	(1.6202, 1.6298)	0	1
Log-normal	$\mu =1.5115$	(1.5115, 1.5115)	85,581	0
	$\sigma =1.6402$	(1.0537, 1.0675)		
Stretched exponential	$\lambda =276.51$	(276.50, 276.52)	813	0
	$\beta =0.0024$	(0.0024, 0.0024)		
